# Interviews with Indian Animal Shelter Staff: Similarities and Differences in Challenges and Resiliency Factors Compared to Western Counterparts

**DOI:** 10.3390/ani12192562

**Published:** 2022-09-26

**Authors:** Deyvika Srinivasa, Rubina Mondal, Kai Alain Von Rentzell, Alexandra Protopopova

**Affiliations:** 1 The Animal Welfare Program, Faculty of Land and Food Systems, The University of British Columbia, Vancouver, BC V6T 1Z4, Canada; 2 Department of Biological Sciences, Indian Institute of Science Education and Research Kolkata, Mohanpur 741246, India

**Keywords:** animal shelter, companion animal, free-ranging dog, India, interviews, occupational health, qualitative research

## Abstract

**Simple Summary:**

Previous knowledge about animal sheltering systems and perspectives of staff working in animal shelters has been centered around Western countries. However, staff in Indian shelters must tackle different kinds of problems, such as care of free-ranging dogs. We conducted interviews with ten participants working in animal shelters in India to begin to gain an understanding of their experiences. Participants reported that inadequate funding, community conflict, and too many animals in need created a challenge for animal shelter work. However, flexibility and positive relationships in their workplace, feelings of duty to animals, and understanding animal needs were identified as positive factors. The perspectives of Indian animal shelter staff showed that certain issues are similar to those encountered in Western shelters; however, other issues are specific to social, political, and cultural influences. Context specific research in animal sheltering is needed to gain a broader world understanding of human–animal relationships.

**Abstract:**

Animal shelters in India are at the forefront of efforts to improve free-ranging dog welfare and tackle animal overpopulation. In terms of cultural and political context, access to resources, and public health challenges, they operate in a very different environment than Western counterparts. Despite these distinctions, current sheltering literature is largely centered around countries such as the United States. The goal of this exploratory study was to examine the experiences of Indian animal shelter staff. Researchers conducted ten semi-structured interviews, in a mix of Hindi and English, with managers, veterinary nurses, and animal caretakers from three shelters. Using thematic analysis, shelter challenges as well as resiliency factors that enable staff to cope with these challenges were identified. Key challenges were inadequate funding, community conflict, and high intake numbers. Resiliency factors included flexibility, duty of care, co-worker relationships, and understanding animal needs. The results of this qualitative study revealed that the experiences of shelter staff are shaped by social, political, and cultural factors and that there is a need for further, context specific research on Indian sheltering rather than only relying on Western perspectives.

## 1. Introduction

In the past 25 years, the goals and activities of shelters in Western countries have changed drastically [1]. Rowan and Kartal (2018) attribute these shifts, in part, to a drastic decrease in animal overpopulation [2]. In 1970, street dogs comprised 25% of the national dog population in the United States (US) and the budgets of humane societies were largely dedicated to population control efforts [3]. Over the last three decades, animal overpopulation in the country has declined significantly, with unowned dogs largely absent from US communities today [2]. This shift has allowed shelters to direct greater energy and resources to other activities, including adoption and humane education.

Rowan and Kartal (2018) suggest that successful shelter practices in the US can act as a useful “template for other countries with large street dog populations”, such as India. However, given that Indian and Western shelters operate in very different socio-cultural and political environments [4], there is a need to examine the extent to which we can ‘export’ sheltering research and practices from the West. With a focus on staff experiences, this study offers insight into sheltering in the Indian context and contributes to the discussions around cross-cultural collaboration, information sharing, and representation in the sheltering field. For the purposes of this paper, we will use the imperfect term “Global South” to refer to countries with low to upper-middle income located in Africa, Asia, Oceania, Latin America, and the Caribbean [5].

### 1.1. Animal Overpopulation in India

Like many countries in the Global South, India faces a significant dog overpopulation problem, with recent estimates projected at 59 million [6]. Free-ranging dogs in the country may experience welfare challenges, including malnutrition, poor skin condition, parasite infections, and human mistreatment, in the form of beating and poisoning [7]. Furthermore, India has the highest number of dog-related rabies cases, accounting for 35% of global fatalities and approximately 20,847 human deaths per year [8].

Until the late 1990s, Indian dog populations were controlled through mass culling, using methods such as poisoning and electrocution. From 1993 to 1999, this “catch-and-kill” approach was gradually replaced with high volume spay-neuter and vaccination programs, with Delhi becoming the first city to implement an Animal Birth Control—Anti Rabies Vaccination (ABC-ARV) facility in 1993 [9]. In 2001, these efforts were formalized under the new Animal Birth Control (Dogs) Rules. This legislation bans the culling, relocation, or removal of street dogs, and requires all state and municipal governments to budget for ABC-ARV work [10]. 

While the ABC Rules identify the government’s responsibility for funding population control efforts, Animal Welfare Organizations (AWOs) are tasked with the actual implementation of these programs. Animal Welfare Organizations are shelters or SPCAs officially registered with the Animal Welfare Board of India [11]. The terms ‘Animal Welfare Organization’ and ‘animal shelter’ will be used synonymously in this paper. 

While many run spay-neuter programs, Indian AWOs are not official regulators of population control. Sterilization practices are government regulated, and outlined in the 2009 Standard Operating Procedures for the Sterilization of Stray Dogs. Under this legislation, Indian AWOs are responsible for using state and municipal funding to run ABC programs “with a standard code of professional practice” [12]. This includes meeting standards for the capture and transportation of dogs, kennel management and ventilation, record keeping, and post-operative care. Thus, it appears that the responsibilities of Indian animal shelters are heavily centered around free-ranging dog care and population control. However, there is an overall lack of peer-reviewed research on the activities of Indian organizations and the experiences of staff in a shelter environment. 

### 1.2. Occupational Health of Animal Shelter Staff

Despite the lack of India-specific research, a broad body of literature has looked at the occupational health of animal shelter staff in other countries. Studies conducted in the US, Canada, Australia, and the United Kingdom (UK) have identified the risk of poor mental health outcomes, such as compassion fatigue and moral injury, amongst animal shelter staff.

Arluke and Sanders (1996) define compassion fatigue as a form of secondary trauma, wherein staff in ‘caring professions’ are psychologically impacted by their distressed patients [13]. Surveying 2879 animal care workers in the US, Hill and colleagues (2020) identified that degree of exposure to cruelty cases was a strong indicator of vulnerability to compassion fatigue [14]. Rank and colleagues (2009) found relationships between the severity of compassion fatigue and the level of involvement in the euthanasia of shelter animals amongst US shelter workers [15]. After assisting with pet euthanasia, staff displayed an elevated heart-rate and lower heart-rate variability. Further, staff directly engaged in selecting which animal to euthanize experienced elevated secondary traumatic stress [15]. Shelter employees involved with euthanasia may also demonstrate higher levels of ‘moral injury’ from engaging in an activity that violates one’s ethical beliefs [16].

At the same time, reduced exposure to euthanasia does not always correspond to improved employee well-being. Andrukonis and Protopopova (2020) examined shelter staff’s experiences in the US. Here, although job satisfaction increased with less euthanasia within animal shelters, the levels of burnout, moral injury, and secondary traumatic stress were also higher [17]. Regardless of the quantity of animal death in a facility, shelter employees are at risk of poor mental health outcomes. Baran and colleagues (2012) studied 102 shelter employees across eight US states, identifying a 27% turnover rate within the first two months of employment [18]. This indicates that new staff may be unprepared for their working environment’s high emotional and physical demands. 

Previous research also demonstrates how staff exhibit ‘resiliency factors’ that help them with the stressors of their jobs. These may include positive shelter activities, social support, and personal attitude [19]. Positive actions, such as community outreach and companion animal adoption, may benefit staff by offsetting the impact of emotionally draining ones, such as euthanasia. Through qualitative interviews with shelter professionals in Florida, USA, Reeve and colleagues (2004) showed that staff benefited from engaging in proactive programs, like adoption drives, and had an increased sense of contributing directly to animal welfare [19].

Shelter staff with strong social support may also be more resilient in their jobs. Amongst 150 shelter staff from Melbourne, Australia, satisfaction with professional and personal relationships was the most significant predictor of euthanasia-related traumatic stress [20]. Employees may also rely on one another to cope with the loss of shelter animals and process challenging cases of animal abandonment or abuse [21]. At the same time, Baran and colleagues (2012) found that US shelter employees who engaged with euthanasia were less likely to discuss struggles outside the workplace, leading them to lose essential forms of social support in their personal lives [18].

Finally, personal attitudes may influence staff outcomes in a shelter environment. Reeve and colleagues (2004) found higher rates of job satisfaction amongst euthanasia technicians with an attitude of acceptance towards euthanasia [19]. Schabram and Maitlis (2017) conducted interviews with 50 animal shelter workers in the US and identified that a staff member’s ‘calling’ (reason for pursuing shelter work) significantly impacted job satisfaction. Individuals who were ‘practice oriented’ (committed broadly to shelter work and animal care) displayed greater resilience than those that were ‘contribution oriented’ (motivated by individual skills and impact) [22]. Holy-Gerlach, Ojha and Arkow (2021) also identified the potential use of social work in animal shelters to reduce or mitigate occupational stress [23].

It appears that animal shelter staff are at risk of a range of negative mental health outcomes from the demands of their jobs. Further, findings on resilience suggest that interventions to mitigate compassion fatigue can focus on increasing positive job activities, social connections, and personal feelings of acceptance to one’s job.

### 1.3. Research Gap and Study Rationale

Existing literature on sheltering and animal care is centered around Western countries, despite significant differences in animal welfare challenges and socio-cultural contexts in the Global North and South.

This may force Indian organizations to draw from research and resources that are not locally relevant. Cultural factors have been identified as a close predictor of the effectiveness of occupational health interventions [24]. Thus, exploring if and where Indian and Western counterparts diverge is particularly important in the context of staff experiences. 

The current study provides initial insight into the experiences of Indian shelter staff through qualitative interviews with managers, animal caretakers, and veterinary nurses. This exploratory research will identify future opportunities for cross-cultural collaboration as well as the areas of Indian sheltering that require context-specific research and interventions. 

### 1.4. Reflexivity Statement

Deyvika Srinivasa is an undergraduate Global Health student at the University of British Columbia. She identifies as being female and of South Indian heritage. Deyvika spent her early years in the US but grew up primarily in Bengaluru, India. Throughout her childhood, she was an active volunteer at animal rescues and community-based spay-neuter programs in her city. Kai von Alain Rentzell is a graduate student in the Animal Welfare Program at the University of British Columbia. He has experience with qualitative research and a specific interest in cross-country dog importation. Kai identifies as male and is of Japanese and German upbringing. He has engaged in companion animal care in multiple contexts through working at veterinary clinics in Japan and volunteering at Canadian dog rescues. Dr. Rubina Mondal (RM) is a postdoctoral scholar at the Indian Institute of Science. She is educated in behavioral ecology and has a particular interest in the welfare and adoption of free-ranging dogs. Rubina identifies as female, lives and works in Kolkata, India, and is actively engaged in dog rescue work in her community. Dr. Alexandra Protopopova, the study supervisor, is an assistant professor in the Animal Welfare Program at the University of British Columbia. Alexandra identifies as female, is of Russian and Swedish upbringing, and has lived and worked in the US, primarily with animal shelters in the south, and Canada. She is educated in behavior analysis and ethology and is particularly interested in animal sheltering and dog behavior and welfare. 

All four authors offered distinct academic and cultural perspectives, which allowed us to adopt ‘insider’ and ‘outsider’ positions as researchers. An insider perspective was developed by having an investigator of Indian heritage (RM) conduct several interviews in Hindi. In this way, staff were able to engage in the research process and share their experiences in a language and with an interviewer with whom they were comfortable. At the same time, our team’s background in American and Canadian animal sheltering placed us in an outsider position. This was beneficial during the analysis process, allowing us to draw cross-cultural comparisons between Western and Indian shelters. However, an outsider positioning may have also led to biases and the interpretation of participants’ responses based on previous researchers’ previous understandings of Western shelter staff. To address these shortcomings, researchers engaged in ongoing self-reflection and implemented a collaborative approach to interview coding and writing. 

The Section 2.5 includes further details about measures taken to reduce bias and improve study rigor. While prioritizing reflexivity, we also recognized the value of a multidisciplinary and multicultural research team and used our different backgrounds to engage sensitively with participants, and eventually draw meaningful and hopefully accurate conclusions from the narratives of Indian shelter staff.

## 2. Materials and Methods

### 2.1. Participant Recruitment and Demographics

Participants were recruited between July and August 2021 through non-probabilistic, convenience sampling techniques (i.e., researchers contacted shelters that had openly available contact information on their websites or social media). 

The study utilized convenience sampling, wherein participants were recruited from three shelters that researchers had already established relationships with. As a result, participants were only recruited from three states (Karnataka, Rajasthan, Himachal Pradesh), and results do not represent sheltering across the country. The impact of a restricted convenience sample is further examined in the Limitations section.

All three shelters had been registered as official Non-Governmental Organizations (NGOs) under the Indian Trust Act for at least eight years. Shelter names and cities have been removed to retain confidentiality, but basic descriptive data collected from shelter managers is presented in Table 1. 

During recruitment, shelter managers were emailed a study flyer that outlined the purpose and format of the research and asked to pass on details to eligible staff. Interested employees were then instructed to contact researchers directly by email or phone. The study flyer included a local mobile number (belonging to the co-author based in India) to ensure easy communication between participants in India and the research team. In the two weeks following recruitment, posters were sent to shelters managers, the authors received replies from ten participants, who reached out directly using the phone or email indicated on the poster. At this point, the shelter managers were informed that recruitment was complete, and we no longer required additional participants at this time.

To be eligible, participants had to be over the age of 18, living in India, currently employed at an Indian animal shelter, and working as a manager, veterinary nurse, or animal caretaker. 

Participants were recruited from three shelters in three different states (Karnataka, Himachal Pradesh, and Rajasthan). 

At the time of the interviews, all ten participants were actively employed and working in person (not remotely) at their respective shelters. The final sample consisted of 10 animal shelter workers (Table 2). Five individuals identified as male and five as female. Participants ranged from 22 to 40 years old, with a mean age of 29.3 years old. Participants held a range of shelter positions: three were employed as managers, three as veterinary nurses and four as animal caretakers. Half of the participants reported their current position was their first formal job working with animals; previous occupational histories for these participants included carpentry, taxi driving, post-secondary education, and family care. Of the individuals with prior animal experience, three out of four had a background in wildlife conservation and one person had worked with a small-scale dog rescue group.

### 2.2. Ethics

The study received approval from the University of British Columbia Human Ethics Board (H21-01759).

### 2.3. Interviews and Data Collection 

The interviews were conducted between July and August of 2021. During recruitment, participants selected an interview time from three to four proposed slots. These times were outside employees’ working hours to reduce the likelihood of staff engaging in interviews at their place of work. If participants were unavailable at these proposed times or only had access to the internet or a phone at the shelter, interviews were scheduled during work hours. Participants were asked if they preferred to conduct their interview in English or Hindi. Six out of ten participants opted for Hindi interviews, all of which were conducted by the same member of the research team (RM). The remaining four interviews were conducted in English by the first author (DS). There was no prior relationship between researchers and participants prior to study commencement. Participants received a consent form by email or text message to fill out prior to the interview and return back. The written consent form informed participants of the specific goals of the research, including an examination of the attitudes and experiences of Indian animal shelter staff.

A semi-structured interview guide was used, divided into three lines of questions: Occupational Health, Dog Management Strategies, and Perceptions of Animal Welfare. The authors aimed to gain a broad understanding of shelter experiences by asking questions on individual, organizational, and societal and cultural levels. The guide contained open-ended questions, such as “Can you talk about your relationship with your co-workers?” as well as closed-ended ones like “Do you feed community dogs?”. The preliminary guide was pilot tested amongst members of the research team and shortened to ensure all questions could be asked in a 60 min period to improve interview flow. The final interview guide consisted of eleven open-ended questions and four closed-ended questions (Table 3). All respondents were asked questions from the interview guide in the same sequence. However, researchers posed additional, unplanned follow-up questions to clarify meaning or inquire about interesting topics raised by participants.

All interviews were conducted virtually over Zoom or phone call and audio only was recorded for transcription. The interview length ranged from 23 min to 1 h 9 min, with an average time of 35 min. At the start of the interview, participants were re-informed of the purpose of the study, and the approximate duration. Participants were also reminded of consent protocols, including their right to leave the interview at any time or refuse to answer questions. Each participant received INR 500 (∼8.55 CAD) as compensation for their involvement. No repeat interviews were conducted due to technological errors or lost data and none of the participants withdrew their data post-interview.

### 2.4. Data Processing 

The second author (RM) and the first author (DS) transcribed, manually verbatim, their respective conversations to generate written transcripts. English transcripts were edited to remove non-standard speech patterns and grammatical errors as laid out by Chang and Spector (2011) [24]. Transcripts were anonymized, to remove participant and shelter identifiers. Interview audio and transcripts were stored on hard drives and computers accessible only to the four members of the research team. Audio files were destroyed after data analysis completion and anonymized transcripts are stored according to university ethics guidelines. 

Transcripts were not returned to participants for correction or comment. However, anonymized interview data was shared with an independent contractor to assess the accuracy of Hindi-English translations. The contractor produced ‘back translations’ of all Hindi interviews by converting English transcripts back to Hindi as laid out by Blauner (1987) [25]. They then compared the back translations with the original Hindi transcripts to identify differences in meaning and flag potential translation errors. All errors were reviewed by the research team. If they were found to be significant (i.e., impacted interview interpretation and analysis), the independent contractor was instructed to listen to Hindi interview audio, re-translate the incorrect section to English and modify the original transcript with the corrected sentence. Overall, back translation analysis revealed there were no significant errors in Hindi to English translations and moreover, that participant responses from Hindi interviews were accurately represented in English transcripts. 

### 2.5. Theoretical Approach 

The present study drew from bounded relativist ontology, which defines knowledge as the common ideas within a ‘bounded group’ (for example, a specific cultural or political orientation) and constructionist epistemology, which identifies that knowledge is generated through interactions between members of this group. Drawing from these foundations, the study implemented an interpretivist theoretical perspective, attempting to situate and interpret participants’ responses in local context and culture [26]. Researchers closely engaged with interpretivism, specifically the concepts of life worlds, situated freedom, and co-constitutionality [27], to design the research focus, methodology and methods, and data analysis processes. 

Given the limited past literature on Indian sheltering and staff experiences, it was not possible to replicate an existing framework before conducting the interviews. Instead, a broad-based interview guide was designed that addressed Occupational Health, Shelter Goals, and Perceptions of Animal Welfare (Table 3). After conducting interviews, all four researchers reflected on the study goal (comparing Indian and Western staff experiences) to select an appropriate research framework. To facilitate cross-cultural comparisons, the team looked to previous literature on Western sheltering and eventually selected a framework from a 2020 study by Levitt and Genzinski, which focused on “Compassion Fatigue and Resiliency Factors” in interviews with American shelter staff [28]. From reviewing interviewed transcripts, researchers identified that participants in the present study placed greater emphasis on broad shelter challenges as opposed to individual struggles, such as compassion fatigue. Thus, *shelter challenges and resiliency factors* was used as the final research framework and aligned with both research objectives as well as participant narratives.

The study used a phenomenological methodology, centering subjective human lived experiences in descriptive data. By applying an interpretive lens, researchers were able connect staff’s lived experiences to a broader social and cultural context. An interpretative phenomenological approach promoted a focus on staff’s overt responses in the interviews as well as their broader experiences in the Indian context. 

The theoretical approach influenced data interpretation and analysis. Researchers specifically incorporated the idea of situated freedom into the analysis process. This concept identifies that while individuals are the ‘experts’ in their own experiences, they are also impacted by their social, cultural, and political environment [29]. When analyzing interviews and identifying themes, researchers centered on participants’ situated freedom and considered how staff may have overcome or been shaped by external forces. This process of ‘situating’ participants’ stated experiences in a broader social, cultural, and political context was crucial throughout the analysis. 

The use of an interpretivist lens in all aspects of the study generated a deep, contextual understanding of Indian sheltering, centered around participants’ lived experiences. At the same time, in line with the idea of co-constitutionality, the results do not represent the only ‘true meaning’ that can be drawn from the interviews, but rather a blend of ideas and interests from participants and investigators. 

### 2.6. Data Analysis

After transcription and back translation, the interviews were analyzed using thematic analysis. Analysis was performed as laid out by Vaismoradi and colleagues (2016), wherein themes are generated through a process of categorizing and summarizing qualitative data [30].

The first author (DS) read all ten interviews multiple times and developed short summaries for each response to a question. The summaries included both basic information provided in participants’ answers as well as interpretation of how this reflects the shelter staff’s attitudes and experiences. For example, the following summary was created based on Participant 5’s response to the question *“Can you describe your role and main responsibilities within your organization?”*:

Participant 5 is involved in a range of shelter operations (treatment, feeding, surgery preparation). She can prioritize tasks depending on the shelter’s needs on a given day and work with a flexible schedule.

The transcripts were then re-read to identify corresponding quotes and evidence for each summary. To assess the level of support for the analysis and establish coding reliability, the summaries were reviewed by the entire research team. All the summarized interviews were reviewed at least once by another co-author. Those with insufficient evidence were either discarded or modified. During this phase, co-authors also identified instances of leading questions and misinterpretation of questions caused by language barriers. The researchers identified one leading question in interviews with Participant 8 and Participant 10. Responses to leading questions were removed from the analysis and not incorporated as evidence for any summary. 

Finally, the summaries for individual questions were compared across participants and used to generate broad themes and sub-themes. Through the entire analysis phase, researchers met weekly to discuss recurring ideas, select important evidence, and refine themes. RM and DS did not make consistent written field notes during interviews but reflected on firsthand interactions with interviewees during group discussions. 

During group discussions, researchers also discussed data saturation and the potential need for additional interviews. Guest, Bunce, and Johnson (2006) define data saturation as the point at which no new themes emerge from the data and no additional data collection is needed [31]. In the context of cross-cultural research, the notion of data saturation can be misleading as it implies ‘outsider’ researchers have understood participants’ experiences to completeness [32]. Thus, researchers in the current study determined sample size based on ‘conceptual depth’. Nelson (2017) defines the latter as the point at which there is sufficient data for researchers to theorize and lays out ten criteria to assess the “sufficiency” of conceptual depth [33]. Data from the ten interviews met “sufficiency” criteria, including clear connections between themes, a wide range of evidence to illustrate themes, and resonance with existing literature. Researchers collectively determined that no further interviews were necessary within the scope of the study. While participants did not provide feedback on the preliminary analysis, they were given the option to receive a copy of the final report.

## 3. Results and Discussion

From the interview responses, the authors identified themes relating to shelter challenges as well as resiliency factors that enable staff to cope with these barriers. Luthar, Cicchetti and Becker (2000) describe ‘resilience’ as the process of rebounding from significant adversity and resultant stress [34]. As stated by Feder and colleagues (2013) identified that this can refer to a range of coping mechanisms, including morals, religion, physical fitness and social support [35]. In the context of literature on occupational health, the term typically refers to protective factors that help staff to mitigate the potential mental and physical health impacts of their jobs. For example, Brintzinger and colleagues (2021) identify ‘emotional openness’ as a resiliency factor against burnout amongst male and female health professionals [36].

The original interview guide included questions on three topics: occupational health, shelter goals and practices, and perceptions of animal welfare. Interestingly, participants’ responses did not divide distinctly across these lines of inquiry. Instead, themes often overlapped in different sections of the interviews. For example, many participants identified that conflict with community members impacted their occupational health as well as their shelter’s practices. Because of this overlap, results were not reported as three separate lines of inquiry, but rather in terms of broad Challenges and Resiliency factors described by staff. For further details on the selection of this research framework, see Section 2.5. 

**Figure 1:**
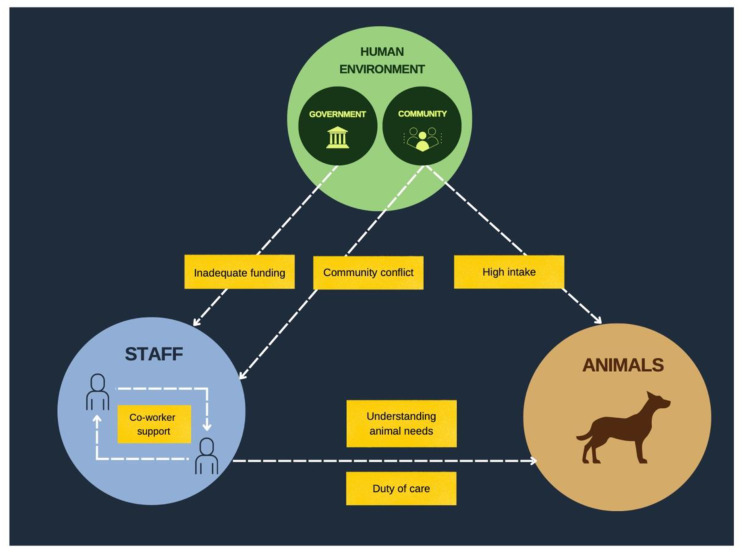
Connections between themes and sub-themes.

In the present study, key shelter challenges were inadequate funding, community conflict, and high intake numbers. In the face of these barriers, resilience factors were the duty of care, co-worker relationships, and understanding of animal needs. The themes and sub-themes are in Table 4. High-level challenges and resiliency factors reflected existing literature on sheltering in Western countries. However, sub-themes, such as government policy, religious beliefs, and a focus on community-based care revealed that the staff’s experiences and assets were also specific to the Indian cultural, societal, and political context. The relationships between themes are sub-themes are detailed in Figure 1.

### 3.1. Shelter Challenges

#### 3.1.1. High Intake 

All participants spoke about the difficulties with managing high animal intake with limited resources. Participants described intake challenges in relation to pet abandonment, animal death, animal overpopulation, and seasonal fluctuations. 

##### A. Pet Abandonment

Participant 1 identified the high rates of abandonment for purebred dogs in India, stating, “The more frustrating part is when people buy breed dogs, pedigree dogs, and they abandon them”. He also highlighted the low outflow of animals from the shelter, explaining that “Local adoptions are not so popular. Nobody wants abandoned dogs”. 

##### B. Animal Overpopulation

In addition to abandoned pets, staff also linked shelter numbers to large free-ranging dog populations. Participant 1 stated, “In India there is an uncontrolled population of stray dogs… [It is] so hard for Indian organizations because there are too many cases, too many dogs that are on the street, that we have to help”. 

Participant 2 identifies high intake as a commonality between Indian and Western shelters, stating, “I do feel like we are similar in many ways. We [both] get a lot of animals in and are all overwhelmed… So, I think that is one thing that really unifies us”. Parallels in inflow patterns are also seen in existing literature in the US, where the intake of both stray dogs and abandoned pet animals to shelters is well documented. Data from the National Council on Pet Populations indicates that approximately 30% of dogs entered shelters as owner-surrenders from 1994–1995 [37], while stray intake is estimated to be 53–83% of shelter dog populations [38,39,40,41]

At the same time, there appear to be distinctions between Indian and Western shelter intake in terms of scale of inflow, the primary source of animals, and reasons behind pet relinquishment to shelters. When asked about the difference between Western and Indian shelters, Participant 2, stated:

A lot of Western NGOs… have quite different situations from what we have in India… We are very [different] in terms of having stray animals as a part of Indian community.

I know it’s not physically possible right now. But I just wish we could reach that stage where all of our dogs are spayed and neutered.

Here, Participant 2 identifies that Indian organizations grapple with a significantly higher caseloads than those in Western countries and may find common objectives, such as population control, more challenging as a result.

Previous literature also indicates distinctions in terms of the primary source of animal inflow. Participants identified that most of their shelter’s work involves the care of free-ranging dogs, through veterinary treatment or sterilization. Participant 2 explained that, when providing treatment for “dogs that are on the street… and part of the Indian community”, staff travel to the animal’s street location as opposed to bringing them into the physical shelter environment. The Pet Care and Facilities Act identifies five primary categories for animal intake (stray, owner surrender, intrastate transfer, interstate transfer, and other) which are commonly used in the design of shelter tracking software [42]. It is evident that free-ranging dogs do not easily fit into these standard categories. For example, the ‘stray’ label includes animals which may have previously been pets (despite having no owner upon intake) and, thus, does not accurately describe an unowned, community animal. This may limit applicability of shelter software in the Indian context. Additionally, there is a question of what counts as ‘animal intake’ as well as ‘outcome’ for an Indian organization, when free-ranging dogs are treated inside and outside the shelter and returned to the community environment. Future investigations are needed into the modification of existing intake categories for Indian shelters and what additional metrics may be needed to track care for community animals. In fact, these metrics may additionally be useful in Western contexts as US animal shelters are moving to community-driven sheltering models [43]. 

Additionally, the inflow of abandoned pets may also be unique at Indian shelters. Participant 1 identifies that many owners “buy breed dogs, pedigree dogs, and they abandon them”. This contrasts with the American context, where most owner-surrendered dogs at shelters are not purebred [44]. In a scoping review, Coe et al., (2014) identified housing barriers, aggressive companion animal behavior, and caretaker personal issues as the most investigated reasons for companion-animal relinquishment at US shelters [45]. The high rates of purebred dog abandonment, specifically, indicate that additional factors influence the purchase, and eventually relinquishment of dogs at Indian shelters. In their ethnographic analysis, Bhan and Bose (2020) describe purebred dogs as a symbol of middle- and upper-class Indian identity, with potential roots in colonial messaging that distinguished British authorities and their “pedigree dogs” from Indian subjects and street dogs [46]. Volsche, Mohan, Gray and Rangawamy (2019) surveyed college students in Bangalore, India, with 89% of the total sample identifying as upper or upper-middle-class [47]. From the total respondents, 62.1% stated they preferred a purebred dog, while only 19.91% preferred Indian street dogs. Preferences for pedigree dogs appear to be connected to Indian class dynamics and potentially influenced by colonial rhetoric. Interventions to reduce pet abandonment at Indian shelters will require an understanding of this interaction between pet attitudes and historical and socioeconomic factors.

##### C. Seasonal Fluctuations

Participants also identified how intake numbers fluctuate throughout the year, increasing significantly during the monsoon (rainy) season, from June-September. Participant 1 stated, “Monsoons are pretty tough because there are lots and lots of cases of maggots and it’s a mating season as well”. Here, she identifies a seasonal spike in the intake of free-ranging dogs for emergency treatment and sterilization. Participant 5 also explains that animal recovery slows at this time:

In the monsoon, healing takes so much time. In our animal birth control program, we have to release the dogs after like 10 days. Otherwise, in other seasons, we can release them after five days because healing processes [are] fast.

With the onset of dog mating, coupled with increased emergency cases, Indian shelters experience larger caseloads from June to September. Western shelters may see similar seasonal fluctuations: Janke and colleagues (2018), for example, report an increase in the admission rate of cats at the Guelph Humane society during spring “kitten season” [48]. Additionally, with global temperatures on the rise, seasonal intake patterns may be subject to change in the coming years. Protopopova, Ly, Eager and Brown (2021) identify that climate change outcomes, such as extreme weather events, are intrinsically linked with sheltering and companion-animal health [49]. The seasonal fluxes at Indian and Western shelters further indicate the sensitive relationship between animal intake and environmental conditions. In the Indian context, specifically, changes in the monsoon season could alter or exacerbate animal inflow. Modelling precipitation and runoff patterns, Clemens and colleagues (2021) indicate that such changes are possible with current greenhouse gas concentrations and project an increase in the quantity and variability of South Asian monsoon precipitation in the next decade [50]. Future studies on climate impacts and mitigation in India should account for their potential effects on both companion animals and sheltering systems.

##### D. Animal Death

Participant 3 describes a slightly different phenomenon; he focused on injured or ill free-ranging dogs, as opposed to abandoned pets, being ‘dumped’ at shelters:

What happens is sometimes people feel that, you know, I don’t want to see those animals dying in front of my house or inside my house… [They say], ‘if I can afford to pay 5000 rupees, I will send her to a shelter, but I will not see the animal dying in the shelter. What happens in the shelter is not my problem’.

Here, Participant 3 describes how many residents are inclined to take injured community dogs to shelters, perceiving them as a haven for animals. Participant 3 describes the contrast between these public perceptions of a shelter environment and the reality that staff experience:

Our Indian ideology is that people think that we will pick up stray animals, put them in a cage and keep them there lifelong by giving them food. But that is not what we experience, no? That is not what we see. [We see] animals dying on us and, you know, it’s very painful at times.

This response reveals the striking volume of animal death to which staff are exposed. It also appears that the public is largely unaware of the reality of overcrowded shelters. Participant 3 expanded on this, describing the typical struggles of new staff at her shelter:

You’re suddenly in a place with a hundred animals who are bleeding, who have wounds, who have maggots in their wounds, who are paralyzed, can’t walk, and whatnot. You’re suddenly in the middle of the room and you’re like, okay, I have to take care of them. It’s not that you are not alone, but you do feel alone.

These insights demonstrate how emergency cases contribute to the intensity of the Indian shelter environment, creating conditions in which staff must cope with an extremely high prevalence of death. The relationship between intake volume and staff wellbeing is well-documented. In a survey of 127 South Australian veterinary nurses, staff with longer work hours and higher contact with distressed clients or animals reported higher levels of work burnout [51]. Additionally, Reeve and colleagues (2004) found associations between seasonal influxes of puppies and kittens and downturns in staff wellbeing [19]. This research suggests that Indian shelter staff may experience greater vulnerability to emotional distress in the busy monsoon months and a reduced capacity to cope with challenging work situations. Leadership training at shelters should address these outcomes, focusing on developing managers’ knowledge of common mental health challenges and their symptoms. This will allow shelter management to identify and extend additional support to vulnerable staff and prioritize positive workplace connections during busy seasons.

#### 3.1.2. Inadequate Funding 

All participants reported funding shortages as a significant barrier at their shelter. Participant 1, a shelter manager, stated, “Sometimes we are out of funds for the dogs, and we have to pay the staff less amount of money”. Participant 3, a manager at a different shelter, expanded on this, describing how these shortages were exacerbated during the global pandemic of coronavirus disease 2019 (COVID-19):

What happened is that the funding that we were supposed to get was all diverted to these COVID activities. A lot of big donors who said they are going to support us, at the last moment, they said, ‘right now I think it’s better to help people rather than animals.

Here, he identifies that, at the height of the pandemic, shelter donors shifted their focus from animal to human support. Participant 7, an animal caretaker from the same shelter, reaffirmed this, stating, “Because of the lockdown, there has been a decrease in the donations”. 

Inadequate funding has also been reported in a Western context: Turner and colleagues (2012) highlight the increasing importance of volunteers in the Canadian context, with many shelters unable to ‘afford’ enough paid staff to deliver animal care [52]. Financial challenges at shelters may have consequences for both animal well-being and the occupational health of staff. Lack of funds may result in the purchase of lower quality food and supplies, worsened facility hygiene, and inadequate animal husbandry due to staff shortages—all of which compromise animal care [53]. Additionally, research on Indian health care staff indicates that wage delays worsen occupational health. Kar and Suar (2014) surveyed nurses across 24 public hospitals in six Indian cities [54]. They found that participants, who reported frequent payment delays, wage cuts, and lack of compensation over time also experienced the highest levels of depersonalization and burnout. 

In the current study, funding challenges at Indian shelters were influenced by three main factors: lack of government support, government policy, and cultural and religious beliefs. 

##### A. Lack of Government Support

Participant 5, a veterinary nurse, “We don’t get any support or help from the government. There are a few locals who help us. But we don’t get any help from the government”. Additionally, the public may lack an understanding of the inadequate support that shelters receive. Participant 3, for example, stated, “Most people think non-profit organizations are getting aid from the administration and the government, but that is not true”.

##### B. Government Policy

In addition to a lack of government funding, government policy may create additional barriers. Participant 3 revealed a specific challenge with securing foreign grants. Here, he references the Foreign Contribution Regulation Act [55]—a set of laws that restrict the flow of foreign funding to the country through tighter registration protocols for both local non-profit organizations and international donors:

The Indian government brought a law saying that, you know, you have to streamline your foreign contribution. That took us a really long time to get all the work done, opening your bank account. So that was again a little painful.

Di Russo (2011) describes the FCRA as the “primary source of the power of the Indian government over volunteer organizations” [56]. This policy lays out the protocol for a non-profit to become eligible for foreign funding and involves a complex evaluation to assess if ‘welfare activities’ benefit local communities. For Indian shelters, successful registration alone may be highly tedious, contingent on registration officers understanding the value of animal shelter work for local communities. Here, Participant 1 describes the 2020 FCRA Amendment Bill which introduced additional control on how foreign funds can be spent once received. These changes add further barriers for shelters and may prevent them from creating a budget that fits the needs of their organization and local communities. 

This tight government control is quite distinct from the regulation of shelters in North America. Past research on the US charitable sector demonstrates that non-profits are under little federal control. With the assumption of their “good faith” intentions, the government relies on non-profits to “police themselves” [57]. This is understandable, given that non-profits in the US are less likely to be beneficiaries of international aid than Global South counterparts [58]. Without the inflow of foreign funds, governments may no longer see the need for regulatory policies, like the FCRA. However, current philanthropic law in the US may also leave room for unethical activity. Milofsky and Blades (1991) for example, describe the insufficient federal direction on recording financial transactions or flagging board member affiliations for health charities in the US and the consequences of unethical fundraising [59].

It is evident that Indian and Western non-profits face varying degrees of government regulation, and that the activity of Indian shelters is shaped by a unique political context. Further, there are broader differences in how shelters in the Global North and South sustain themselves: while the latter can rely on domestic financial resources, Global South countries, like India, appear to access both international and local funds.

##### C. Cultural and Religious Beliefs

Beyond government barriers, participants noted difficulties with gaining community donations. Dog rescues may struggle to gain local support because of the greater cultural and religious importance of large animals in Hindu communities. For example, Participant 1 stated, “We don’t take large animal cases like cows and donkeys and all that stuff. People really don’t want to donate for dogs. That’s why we have very few donations”. Participant 4, an animal caretaker at the same shelter, also spoke to the prioritization of large animal welfare and push back from locals when the shelter is unable to house cattle and goats:

Many times, people accuse us [of not doing our jobs]. We tell them that yes, we are an animal rescue, but we don’t have space to keep large animals. We can treat them, but we can’t keep them. It feels bad to tell them that.

Participants 1 and 4, who lived and worked in the Northern states of Rajasthan and Himachal, respectively, both describe community members’ frustration that shelters prioritize free-ranging dogs over the care of large animals, such as cows, buffalo, and donkeys. Large animals in India are part of a complex “cultural ecology”: buffaloes and donkeys hold economic importance in the country’s largely agricultural economy, while cows have great religious, social, and political significance [60]. Chigateri (2008) explores the intersection of religion and attitudes towards animals, highlighting the perceived sacredness of cows amongst dominant-caste Hindus [61]. Parikh and Miller (2019) explain how this narrative has been harnessed by political actors to subjugate minority Dalit and Muslim communities [62]. In this context, a shelter’s ability to gain local support is not simply a function of the quality of care but rather whether the ‘correct’ animals are being cared for. Interventions to increase donations will require sensitivity given the religious and cultural standing of different species of urban animals, as well as the complex history of human race and ethnicity in India.

The relationship between religion, culture, and donations at Indian shelters illustrates another potential divergence from Western counterparts. Surveying communities in the US, Wang & Graddy (2008) found little relationship between religiosity and charitable behavior: though participants were more likely to donate to their specific religious group, they were no more likely to donate to secular causes [63]. Though little work has been done on identity and donation to animal causes in Western countries, religious dimensions do appear to be more significant in Indian sheltering. This indicates that any successful community engagement and funding strategies must account for these nuances and cannot merely replicate those used in Western organizations. 

The current study was constrained to religion in the Indian context; Hindu communities in other countries may offer a different perspective. Past research has examined religion and animal attitudes in Bali, Indonesia, where over 80% of the province practices Hinduism [64]. Analyzing community perspectives towards dog meat consumption, Corrieri and colleagues (2018) identify how tenets of Balinese Hinduism have shaped and often promoted animal welfare in the country [65]. This includes the concept of “Pale Mahan” [harmony with one’s natural environment] which encourages equal appreciation for all animals, including livestock, pets, and community dogs. Surveying residents across ten Balinese villages, Widyastuti and colleagues (2015), identified how Hindu beliefs might impact the treatment of free-ranging dogs [66]. When asked why they would not kill Balinese street dogs, residents cited the Hindu principle of ahimsa (non-violence).

Additionally, religious beliefs may not entirely dictate the treatment of animals. While Hindu principles prevented community involvement in dog culling, participants did not oppose the discarding of unwanted female puppies near garbage dumps or waterways [67]. In Balinese communities, positive religious attitudes towards dogs did not guarantee welfare-promoting behavior. Similarly, in the Indian context, while Hindu communities may promote cow welfare, individuals could act against these norms. When examining how religion affects Indian shelters and their funding, it is important to recognize how complex combinations of social factors, including, but not limited to, religion, impact human–animal interactions.

Participants in the current study demonstrated that, though financial instability is typical in animal welfare work, their experiences were shaped by a combination of religious, cultural, and political dimensions. A deep understanding of this complex local context is crucial to effectively tackling funding challenges at Indian shelters. 

#### 3.1.3. Community Conflict

Conflict with community members was another key challenge reflected on by participants. Participant 3, a shelter manager, explained the impact of clashes with the community: “See, handling animals is very easy, but handling people is very, very tough [laughs]. So that is something that really, you know, that takes a toll on you”. These conflicts were of three main types: rescuer pressure, resident pushback, and incorrect community care.

##### A. Rescuer Pressure

All three shelter managers identified struggling with the large volume of calls from rescuers. Participant 1 stated: The biggest challenge for me is the helpline that I manage here. The people call up for rescues and many other things. I have to properly deal with them, make them understand. Participant 2 also explains her distress when sorting through these requests, identifying that “Every day we’re getting around a hundred complaints to prioritize… Whom do you need to reach first? Who will die?”. There seemed to be a lack of understanding amongst the public about these experiences and the sheer volume of requests shelters receive. Participant 2 speaks to this issue, stating, “The other person just sees oh, the ambulance could not reach. Whereas we are actually sitting in between a hundred calls and going ‘Oh god, what do we do?’”. Beyond the volume of requests, staff also navigated difficult conversations with individual community members. When asked to recall a recent challenging case, Participant 3 described the following:

There was this one scenario I still remember. There was a dog with a broken pelvic bone—the pelvic was broken into almost three to four pieces. So, there was no way to repair that dog… But the rescuer said, ‘No, I don’t want to euthanize this animal.’ She said she’d like to take it to some other place. So, she took the dog, did the surgery, and the dog died on the table.

Cases such as these, where an animal’s life is at risk, emphasize the emotional burden placed on staff when speaking with community members. Participant 3 highlighted the intensity of their jobs’, stating, “You have to deal with people who bring in those animals and sometimes it’s a lot of emotions, you know? The working environment in a shelter is never not stressful”. When asked about how community members impact her on a personal level, Participant 2 explained that “When somebody loses their animal, who is super, super attached to it. You get a load of people who are coming in and saying that you did not do enough”. 

##### B. Resident Pushback 

In contrast to rescuers who blame staff for ‘not doing enough’, local residents may also oppose any shelter activity in their area. Participant 5 describes this challenge:

There are some people who do not like shelters… Sometimes, if we have to catch a dog, people will chase it away. They will not tell us where the dog is or if there is any problem or if they have to put in some effort.

Participant 3 also identified pushback to mandatory spay-neuter protocols at his shelter, stating, “A lot of time people are against it, but we tell them, this is mandatory”. Despite receiving pushback, all shelter staff were willing to uphold shelter policies relating to animal birth control, even if it resulted in conflict with community members.

Shelter staff in Western countries appear to navigate very similar situations. Loyd and Miller (2010) surveyed Illinois homeowners and identified that most participants opposed their local shelter’s TNR (trap-neuter-return) programs for controlling feral cat populations, favoring relocation of the animals instead [67]. Ashforth and Kreiner (2014) describes the stigmatization of shelter staff from wider communities and social construction of aspects of shelter work, such as euthanasia, as ‘dirty work’ [68]. Lopina and colleagues (2012) identified that such perceptions may heighten burnout and emotional strain amongst shelter staff. Interestingly, participants in the current study reported positive reactions from friends and peers to their jobs and a lack of community stigma. This is understandable given that the moral and physical ‘taint’ is most associated with the high-volume convenience animal euthanasia at Western shelters [69]. Additionally, Mendonca, D’Cruz and Noronha (2022) identify how ‘dirty work’ stigmatization for Indian cleaning workers may intersect with caste and class stigma; further investigations can consider whether the stigmatization of Indian shelter staff is similarly impacted by social position [70].

##### C. Incorrect Community Care

While there were challenges with residents who oppose shelter activities, Participant 3 also described issues created by individuals who provide incorrect care to community animals. He specifically identified the challenges with residents who feed free-ranging dogs:

And there are a lot, a lot of unethical feeders. So, yeah, rather than solving any issues, it creates a lot of problems: These people are keeping them with milk and rice and non-veg. Milk and rice will give them loose motion. So, the dogs are going to be pooping near all these peoples’ houses and no one will feel comfortable to clean it after feeding them. [People in the community] say: ‘You know what? You take them to your house, look out for them in the house. Don’t feed them here. We don’t want these dogs here. So, there’s a lot of conflict.

Here, Participant 3 identifies food, such as meat or eggs (“non-veg”), and milk and rice, that are commonly fed to free ranging, but potentially unsuitable for them. The manager sees a connection between unethical feeding, increased dog disruptions in the community, and heightened ‘anti-dog’ sentiment from residents who do not feed. Participant 3 also highlights the negative impact of incorrect feeding practices on free-ranging dog welfare:

[And] you know, not like three meals a day, if you’re feeding them, feed them every alternate day, because the animals shouldn’t be dependent on one particular person. So, when you start feeding them on a daily basis, you are killing their survival instincts. You know, it becomes very difficult for the animals to survive.

According to Participant 3, the role of a feeder is to supplement community dogs’ diets, without making them entirely dependent on human support. It seems that many residents struggle to strike this careful balance. Participant 3 further identifies that with a strong focus on daily feeding, other important activities may be neglected. He stated, “When you feed a stray dog, you need to take the responsibility to make sure that the animal is sterilized and vaccinated”. While residents are eager to engage in low-barrier forms of care, such as feeding, they may be more reluctant to help coordinate sterilization and vaccinations programs that are crucial for long term animal welfare. 

Here, it is apparent that Indian shelters must strike a careful balance between encouraging the feeding of community dogs while allowing them to retain independence and, in this way, performing an educational role similar to a wildlife rescue. Indian shelters must engage community members in nuanced discussions of the needs of free-ranging dogs. In contrast to Western shelters, who operate in areas with lower numbers of or no free-ranging dogs, Indian shelters may navigate a more complex set of responsibilities and community conflicts. 

The parallels between challenges at Western and Indian shelters, in terms of funding, managing overcrowding, and navigating community conflicts indicate the potential for greater collaboration and information sharing on population control efforts and community engagement between the two countries. At the same time, context-specific factors, such as religious influence, government funding policy, and free-ranging dog feeding, highlight the unique barriers faced by Indian staff and need for context-specific interventions.

### 3.2. Resiliency Factors 

In addition to identifying shelter challenges, interviews also revealed important resiliency factors that allowed staff to cope and succeed in their jobs. Researchers divided the resiliency factors into four main categories: flexibility and prioritization, co-worker support, duty of care, and understanding animal needs.

#### 3.2.1. Flexibility and Prioritization

All participants displayed an immense amount of adaptability. Amongst animal caretakers, this was seen through their comfort with varied working hours and responsibilities. Participant 9, for example, stated, “It’s not fixed working hours… For three to four weeks, I worked in the 7-4 pm shift. Then 10-7 pm for four weeks. Now I am working the night shift”. While Participant 10 described the fluctuations in her day-to-day tasks:

If I am doing some work, like if there is some priority case, then we handle them first. If someone’s clothes [i.e., bedding/bandages] are wet, we change them immediately. If they need hot water, we get it done… If someone hasn’t eaten, then we retry feeding them. If someone needs an extra egg, we give them to ensure that the feeding is complete. If someone’s clothing is dirty, then we change those.

Rather than sticking to a rigid set of protocols, Participant 10 was capable of monitoring her environment and making decisions to optimize the comfort of shelter animals. From Participant 1’s responses, we saw that adaptability is also important in a management role. Despite having a range of administrative duties, her priorities were very similar to that of animal caretakers - tasks directly related to animal care were placed at the top of her list:

I am that sort of person who ends up taking more on her plate than she can manage, even if it’s just going and checking up on somebody and spending 15 min there and I’m like ‘Oh god I could do something else!’, but that was important for me at that particular point in time.

Flexibility was also seen in staff’s response to emergency cases. Participant 10, for example, described how her shelter contacts other frontline groups to facilitate large animal rescues: “Sometimes we contact the fire brigadiers. They send over a team. They are already trained for large animals. So, for large animals, they come when we ask them to”. Participant 4, a veterinary nurse, described similar collaboration in a veterinary context:

No ma’am, we don’t have an X-ray machine. We go to a private clinic for those. There are some in [shelter city]. We have a CBC [Complete Blood Count] machine now. Any other biochemical tests are done in private clinics.

Participants’ ability to adapt to fluctuating schedules, caseloads, and resource limitations, may indicate their high levels of ‘psychological flexibility’. Kashdan & Rottenberg (2010) describe psychological flexibility as the human ability to adapt to situational demands, shift behavior, and remain open to new mindsets [71]. Previous literature has established the positive effects of this mindset on shelter staff. In a study on 170 non-profit service works, Biron and van Veldhoven (2012) found that personal psychological flexibility was associated with reduced emotional exhaustion as staff were inclined to accept, as opposed to repress, their emotions [72]. Psychological flexibility appears to be a powerful indicator of both well-being and work performance. This mindset may help staff to mitigate previously identified challenges, such as conflicts with rescuers and residents, and, in turn, prevent emotional exhaustion. 

#### 3.2.2. Co-Worker Support

##### A. Collaboration 

The sentiment of collaboration was also seen amongst co-workers. Participants saw relationships as incredibly important for their wellbeing and described having an intimate, family-like environment at the shelter. Individuals that had moved to a city, from rural areas, to pursue employment had particularly deep connections, based on their shared backgrounds. Participant 4, for example, stated, “We are from the same village, four of us boys. We are from the same village, so it feels good to work together”. Participant 4 also had positive interactions with higher level management and explained, “My boss is also good. Quite good. So, it’s fun to work.” Participant 9, another animal care worker, even noted an absence of rigid hierarchies in his organization: “Nobody thinks that he is a worker, he is a compounder or doctor, there is nothing like that. We speak to each other lovingly. They call me [omitted], my name”. This significance of workplace friendship was also seen in management. Participant 2 described relying on her colleagues for social connection given the amount of time she spends at the shelter:

So, nine hours of working plus like about two hours of traveling every day… It almost consumes my entire life. So, it’s like, my coworkers are the entire family and friends I have, my life is very sad [laughs].

These informal, family-like relationships seemed to have positive effects on the working environment. Participant 5, for example, explained that she trusts that her co-workers will support her and stated, “If there’s any time that there’s too much work and I am unable to handle it, I can go and tell someone that I am unable to handle or finish this job and ask them for help to do it. So, it gets managed”. Participant 2 described her collaborative decision-making approach as a manager:

If things are being changed then, I want them to understand that it’s for the bigger animal welfare picture. I try to explain to them why a certain decision is being made. Or if I’m scheduling them somewhere, then why is it so important, why them and not somebody else.

Beyond including employees in decisions, Participant 2 also prioritized providing emotional support to staff:

[I try to be] emotionally available for [new staff], because this [work] is so overwhelming… So, we try to gradually and slowly move them forward, and also be there and try and talk to them as to how they feel about it. I’m always trying to always find a balance where people can be able to express themselves and not get overwhelmed.

##### B. Equity and Safe Space 

Managers also offered nuanced and individualized forms of support, with the goal of improving staff’s equity and autonomy both in and outside the shelter. Participant 3, for example, explained how she created safe spaces for her female employees:

A lot of women that we get from the local villages have so much responsibility. They need to go back home and cook for their husbands. And sometimes they are not in the best situations. So, I really want to make these women feel more comfortable, not just in their workspace. But also, that it’s okay to say, ‘I’m not in a good place at home’.

If they’re going through something at their home place and you see that someone is down, like their energies are not as they used to be, we try to talk to them sometimes and see if we can help them out sometimes. Because it’s already too much to go through in the workplace—we are continuously stressed and you’re working nine hours a day. And then you go back home, and you have another issue.

Participant 2 put her views on equity into action by implementing with tangible structural changes, such as promoting female staff to positions of authority:

I would say about 35 to 40% women and then the rest of them are men. It’s still predominantly men, but the shelter area is handled by women. [Name omitted] and [name omitted] two of our very strong women, they’re like the best caregivers that we have. Any new staff who enters the shelter, irrespective of their gender, needs to know that both of them are their bosses.

It’s also important to make them feel empowered. You are working. It’s you who is running the family. You are as independent as a man out there. So don’t, in any area, feel like you don’t do enough or feel like you are obliged to something.

Participant 2’s approach to management, focused on promoting staff’s professional and personal wellbeing, is quite distinct from shelter governance in North America. Yoffe-Sharp (2012) examined the culture in US humane societies, identifying rigid hierarchical operations that may heighten staff conflict, worsen communication and create perceptions of unfair treatment by management [73]. By contrast, Indian non-profits may avoid stringent professional norms when interacting with co-workers. Sharma and colleagues (2019) surveyed 100 non-profit employees in Jaipur, Rajasthan, identifying that job satisfaction increased with informal co-working gatherings and comfort with supervisors [74]. The same seems to apply to Indian shelters, with staff prioritizing intimate connections with one another and acknowledging the interconnectedness between personal and work challenges. In doing so, an environment of genuine care is created that helps staff cope with the chronic stressors of their jobs.

#### 3.2.3. Duty of Care

In addition to a commitment to coworkers’ wellbeing, participants also demonstrated a deep sense of duty towards animals. All staff saw their work for animals as an essential service, found ways to ‘manage’ high shelter intake, refusing to turn animals away, and extended care to community dogs outside their working hours. 

This mindset was seen in Participant 1 who, after describing overcrowding at her shelter, stated, “We cannot neglect any rescue, [by] saying that we don’t have space. We have to manage”. Participant 4 reflected a similar sentiment. After explaining that there is “no space” for new rescues, he quickly emphasized, “We cannot refuse calls for large animal rescue either”. 

A duty of care mindset is also reflected in the high quality post-operative care protocols for sterilized animals. Participant 10, an animal caretaker, stated, “Our shelter has facilities for their stay, food and water, and good care. Some can stay ten days, some five days. Meaning, till the animal requires time to get better”. Participant 5 reflects a similar practice at her shelter, stating, “If any animal is very weak, then we first nurse them back to health. Then we do the surgery and only then release it back”. Despite dealing with a very high volume spay-neuter program, staff appear committed to the individual recovery and welfare of sterilized animals. 

Non-negotiable care was also extended to community animals. For many participants, caring for free-ranging dogs outside of their work was part of their daily routines. When asked about this topic, Participant 2 stated, “I have nine dogs that I take care of every single day. They sleep in my house. I get beds for them”. Participant 1 also reported caring for many local animals but focused on feeding dogs on the streets as opposed to sheltering them in her home. She stated, “I have 32 stray dogs with me that I have rescued myself. So, whenever I see a dog and I’m feeding and they come outside my gate, yes, I feed them. I love to feed dogs.” 

Some staff have family members who also care for community dogs. Participant 8 described feeding dogs along with her spouse:

Me and my husband, daily we feed around 30 dogs. After we come back [from work], all the dogs are there. ‘When they come, when they come!’ They are waiting for their meal [laughs].

In contrast to staff who fed daily, some participants explained that they simply extended care as needed. This was the case for Participant 6, a veterinary nurse:

Yes, I feed them sometimes. For example, if I come across some dogs on the road and they approach me, I give them something. And if I know some dog, especially the dogs suffering from mange, you see a lot of mange-infested dogs around, so for treating them I usually put the tablets in some food and give it.

While the exact type of care varied, all participants had strong emotional connection with their community animals. Participant 9, whose neighborhood dogs appeared to trust and have a strong bond with him:

When I return, they get very happy. Sometimes they start fighting on seeing me or during feeding. They otherwise usually don’t fight among themselves… The moment they see me, they come to me running.

Further, staff did not view feeding as a burden on top of shelter duties. In fact, Participant 1 identified that her shelter role put her in a good position to care for community animals:

I have made them different kennels, so they stay in their kennels. Every day I pick up their poop and all that stuff because I’m used to it, because I work in an NGO and it’s my daily work.

It seems that many staff felt their jobs made them an asset to the community. Participant 4, a veterinary nurse, expressed his willingness to provide treatments outside the shelter, stating, “I have told them [hotel staff] that you are doing a very good job feeding them [dogs]. If there’s any problem with any of them, call me, I will personally come to treat them”.

High exposure to animal suffering, animal death, and large volumes of stray animals appear to increase shelter staff’s vulnerability to compassion fatigue [15]. Despite being exposed to many of these risk factors, participants appeared to be highly resilient and did not indicate overt symptoms of compassion fatigue. This may stem from the ‘duty of care’ mindset: by viewing their jobs as an essential service, staff may feel an increased sense of pride and fulfillment, even under challenging circumstances. Additionally, having a sense of ‘duty’ towards animals places them in a position of autonomy, with the ability to take action and improve animals’ outcomes. This is in contrast to experiences of euthanasia technicians in the US, who may experience feelings of helplessness from the requirements of their jobs [13]. With a greater sense of feelings of duty and control, Indian staff are perhaps more resilient when faced with similar stressors in their jobs.

Of all ten participants, only Participant 3 identified a hesitancy to care for community animals, citing fears of being ‘harassed’ by local residents:

To be very, very honest, I don’t feed any animals in my neighborhood. The reason is because, what happens is when I start feeding them people will start asking me or there have been cases where people will dump animals into my house. So, when they know that I’m associated with an association like this, they’ll be like you know what, take away this dog. So, it becomes a huge problem for me and for my family members.

People will ask you for medication, people will ask you for breed dogs, where do you get it, what do you do, how to get rid of this dog, cat. Answering all of these queries sometimes is really very stressful.

While a sense of duty towards animals may connect shelter staff to their work, Participant 3’s responses also highlight the dangers of this mindset. When the protection of all animals in their shelter and home environment is seen as non-negotiable, staff may be unable to draw boundaries and combat feelings of overwhelm and stress. 

#### 3.2.4. Understanding Animal Needs

In addition to engaging in animal feeding and care, participants differentiated between free-ranging and pet dogs, and reflected on their unique needs. While staff identified the importance of human care for dogs’ physical health, they stressed that animals’ emotional wellbeing—that is, what they need ‘to be happy’—was maximized with greater autonomy and reduced human intervention. Adopting a nuanced perspective, which acknowledges physical and emotional experiences, allowed staff to identify their specific responsibilities to community animals, while also acknowledging limits of their support. Participants specifically identified unrestricted movement, autonomy, and community care as the most important ‘metrics’ for free-ranging dog welfare.

##### A. Unrestricted Movement

All participants identified unrestricted movement as the most important aspect of good welfare for free-ranging dogs. Unrestricted movement referred to the ability of free-ranging dogs to move freely in their neighborhood, independent of human control. Participant 4 described his fears that free-ranging dogs would be uncomfortable if treated as traditional pets:

Sometimes if we get them adopted, then they [the dog] starts wondering, why have I been restricted. For example, if we are suddenly asked to leave our house and start staying somewhere else, we will also feel odd and face issues.

Expanding on these ideas, Participant 8 recounted her personal experience with keeping free-ranging dogs in her home, stating, “One time I put them in my compound, they were very afraid. They felt uncomfortable. Now, they want food two times a day and they feel happy.” By referring to their happiness and need for space, it is evident Participant 4 considered the animals’ physical and emotional state as part of their overall welfare. Participant 5 expands on these ideas, identifying, “The street dogs that are there, they have a life. They like to stay open, unrestricted”. She further draws a distinction between free-ranging and purebred abandoned dogs:

Abandoned dogs cannot survive outside. They have no idea how to walk on the road, where to get food, water. They have no idea about anything. So, we should definitely try from our end to find them homes, good homes.

Distinguishing them from typical pet dogs, which require direct care and supervision from their owners, Participant 5 emphasizes that free-ranging dogs are highly robust and able to live independently, without human control. 

##### B. Autonomy

The idea of autonomy was also connected to positive welfare. Participants described the community dogs as capable of making independent decisions. Participant 2 spoke to this idea:

My [community] dogs have the best living situation as then they can go around and chase whoever they want. My home is forever open for them, so they can walk in whenever they want, and they can walk out wherever.

Here, Participant 2 emphasized the importance of the animal ‘choosing’ the human and making a conscious decision to return to their home. Participant 4 described a similar relationship with his community dogs, who are not kept in his house, but come back to him willingly:

We don’t tie up those dogs, so they roam around. We have a lot of open space here. They know they will get food in the evening. They come back at that time.

He identifies that community dogs can actively make decisions, informed by patterns in their environment, without directions by a human owner. Participant 6 extends the idea of independence to social behavior:

I think these dogs can be kept at home, but they are street dogs. They should be allowed to roam out as well as allowed to stay inside the house. It shouldn’t happen that the dog is kept inside the house 24 × 7 and only sees the humans of that house. They should mix with others too.

Here, we see the importance of varied social interactions for free-ranging dogs and their ability to forge relationships autonomously. Participant 6 identifies that community dogs thrive on interactions with both humans and conspecifics and thus, need to be able to move independently in their environment to form these varied social connections.

##### C. Community Care 

Finally, many participants highlighted the importance of community-based care to ensure the welfare of free-ranging dogs. Participant 6 stressed that local residents can easily reduce injury and harm to community animals, stating, “They will have a better life on the streets ma’am. If people drive a little more carefully and if it’s [the dog] taken care of, then they will be happier on the streets”. Participant 6 further identified the potential involvement of community members in medical care and animal birth control programs. He suggested that a decentralized animal care system may ease the burden placed on animal shelters:

Some dogs may be taken care of by the locals. If something is wrong with them, the medicines are handed over to their local caretakers. Then there is no need to send them to the shelter… We need to make the local people aware of ABC and sterilization and that they can go to any shelter/NGO to get it done. Or if they are having trouble, then they can gather a few people for help and go to a government hospital and get that done.

This notion of community-based care was also seen in conversations with Participant 2. When asked if free-ranging dogs can have a ‘good quality of life’, she stated:

I absolutely disagree to say that if they’re living on the streets, then they don’t have a good life if they have people in the community to take care of them. As long as these dogs on the street are community dogs, dogs that the entire community takes care of. I don’t see an issue in it.

She further highlighted the power of collective actions, encouraging each community to take responsibility for a few animals in their locality and identified the importance of the first step of ‘getting to know’ your neighborhood dogs:

If a community does decide to take care of these dogs, they don’t have to take care of like a hundred dogs. They know that these nine dogs will stay in my lane. So, they will develop a relationship with these dogs because they stay there, and they know these dogs. They know, this one eats a lot. You know all those small details.

In relation to dogs’ physical health, participants acknowledged the role of community members. However, when asked about the animals’ emotional wellbeing, staff stressed the autonomy and unrestricted movement. This perspective, which promotes *reduced* human intervention, contrasts attitudes to animal welfare in the West. Tuan (2003) identifies that Western human-dog relationships are based on a combination of domination and affection: owned dogs are constrained physically within a home and restricted socially to a specific owner [75]. At the same time, they receive intense attention and love, often becoming integrated into the human family [76]. These perspectives have resulted in a rigid culture of ‘responsible pet ownership’ in the West, where to be a good owner is to be always in complete control over one’s animal [77]. Additionally, Haraway (2003) identifies that pet-keeping standards inform dog welfare in other contexts, including shelters [78]. Thus, shelter staff may be under pressure to control and care for animals as pet owners do and feel disappointment if they fall short. Such experiences were documented in a 2017 study by Schabram and Maitlis, where emotions of shame, guilt, and personal disappointment were seen in narrative interviews with 50 shelter staff [79]. Indian shelter staff offer a different perspective, by valuing animal autonomy but also recognizing limits to which human caretakers can enhance welfare. Staff see themselves as a source of support, rather than control, for animals and, in this way, may relate more positively to their jobs and performance. A similar perspective, if applied in the Western context, may allow shelter workers to feel more successful and empowered in a demanding environment. 

While animal attitudes vary between cultures, they may also fluctuate within them. In the present study, participants saw native free-ranging dogs as being independent but expressed that abandoned purebred dogs “cannot survive outside” and require “good homes”. Their understanding of pet dogs resembles Western attitudes on animal ownership and control. It appears that the emphasis on autonomy was not applied ubiquitously to all dogs, but rather specifically to community dogs. Fluctuations in animal attitudes are also seen in the Western context: Jorgensen and Brown (2014) investigated leash-law on public beaches amongst pet owners in McConaughy, Nebraska and found that less than 25% of owners abided by regulations, despite expressing negative beliefs about unleashed dogs [79].

It is apparent that attitudes to animals are neither universal nor consistent within “cultural landscapes” [80]. Yet, there appears to be inherent value in understanding the different ways in which humans relate to animals and looking beyond dominant Western rhetoric [81]. The resiliency factors identified in the current study demonstrate the unique ways in which Indian shelter staff relate to animals and humans in their environment: staff prioritize connections with co-workers and shelter animals and appreciate the independent relationships amongst community animals. This relationship-based coping may inform effective support interventions for shelter staff beyond the Indian context. Despite extensive evidence of compassion fatigue amongst Western shelter staff, administrators are often unprepared to provide support due to a lack of knowledge about cost and time effective interventions [18]. Past research has focused on external interventions, such as skills-based training, counselling, and stress and coping seminars to address compassion fatigue. This study suggests that a community and relationship-based approach, as exemplified by Indian staff, may have long-lasting effects on emotional well-being.

## 4. Limitations and Future Research

There were several limitations in the study that must be acknowledged. Participants were selected from only three states (Himachal, Rajasthan, and Karnataka). As a result, our results do not reflect the diversity of cultural experiences and animal care across Indian states. In the present study, participant recruitment was restricted as the research team was only able to conduct interviews in Hindi or English. Future projects can prioritize collaborations with translators who are to facilitate interviews with shelter staff in their local language. 

There may be general challenges with conducting reliable trans-linguistic interviews. In this study, the translation of Hindi interviews to English was performed by one member of the research team and an independent contractor was used to identify any translator errors. Lopez and colleagues (2008) propose a more rigorous protocol for conducting reliable cross-cultural research. The authors describe a seven-step methodology which includes the use of multiple independent translators, and group discussions about variations in meaning based on region and dialect [82]. Future studies, particularly if facilitating interviews in a larger number of Indian languages, should implement a similar process to ensure translations represent participants’ experiences as accurately as possible. 

In the present study, the use of audio recordings alone may have limited the depth of the results as researchers were not able to record the subtleties of non-verbal expression. Furthermore, conducting interviews over Zoom may have impacted participants’ responses if they were uncomfortable with using an online platform. Repeating this study with in-person interviews would allow researchers to pick up on the participant’s body language and remove any barriers created by technology. 

While this qualitative study offers initial insight into Indian sheltering, large-scale quantitative research may be needed to see whether the discovered themes represent overarching challenges and resiliency factors in a representative sample of Indian animal shelter staff. This methodological triangulation would allow for a more comprehensive understanding of staff experiences and improve the credibility of the current findings. This may be important when considering the impact of government policies, such as the FCRA, that regulate foreign funding. Future surveys can identify how many Indian shelters rely on foreign funding and are, in turn, impacted by such legislation. Additional investigation into the psychological experiences of shelter staff is also needed. Many participants were reluctant to discuss mental health and the emotional challenges of their jobs and denied experiencing any burnout or compassion fatigue. While this may reflect staff’s resilience, it may also be the result of cultural stigma around mental health and openly addressing one’s struggles. In future research, implementing a mixed-methods approach (by posing questions about mental health in an anonymous survey format as opposed to an interview) may make Indian staff more comfortable, if they were not, in opening up about the emotional experiences in a shelter environment.

## 5. Conclusions

While past literature has largely focused on the Global North, this study demonstrates the importance of recognizing Indian sheltering as a distinct area of interest. This is demonstrated by challenges, such as government regulation and cultural preferences for large animal care, that are specific to the Indian context and impact a shelter’s ability to sustain animal care operations. At the same time, the identified resiliency factors indicate that Indian staff also cope with job stressors in unique ways. Participants in this study may harness relationships with both animals and humans to increase resilience and maintain their mental health. This ‘relationship-centered’ perspective can be applied to the Western context to design preventative measures against compassion fatigue that focus on deepening staff’s connection with one another and the animals for which staff care. Furthermore, others can use insights about the identified needs of dogs, such as a need for autonomy, to reduce any potential ethnocentric biases in the determination and improvement of animal welfare. While such cross-cultural knowledge exchange may be very powerful, this study also highlights the need for far more research focused specifically on the Indian context. Further studies should investigate the specific challenges and staff experiences at Indian shelters and how socio-cultural and political factors influence the capacity to support both human and animal welfare.

## Figures and Tables

**Table 1:** Animal Shelter Characteristics.

Location [State]	Primary Animal Types	Total Number of Employees	Annual Animal Intake
Karnataka	Dogs, Cats, Rabbits	44	400
Himachal Pradesh	Dogs	#	1500
Rajasthan	Dogs, Cats, Cows, Bulls	100	11,182

# Data unavailable.

**Table 2:** Participant Demographics and Employment Data.

Participant	Job Title	Gender	Age	State	Interview Language
P1	Manager	Woman	31	Rajasthan	English
P2	Manager	Woman	26	Rajasthan	English
P3	Manager	Man	27	Karnataka	English
P4	Vet nurse	Man	32	Himachal Pradesh	Hindi
P5	Vet nurse	Woman	27	Himachal Pradesh	Hindi
P6	Vet nurse	Man	28	Rajasthan	Hindi
P7	Caretaker	Man	22	Karnataka	Hindi
P8	Caretaker	Woman	#	Karnataka	English
P9	Caretaker	Man	31	Rajasthan	Hindi
P10	Caretaker	Woman	40	Rajasthan	Hindi

# Data unavailable.

**Table 3:** Interview Guide.

Occupational Health
Can you describe your role and main responsibilities within (stated) organization? What does a typical day of work look like for you and what are your working hours? Can you talk about your relationship with your co-workers or supervisors? Do you generally find the workload manageable? **[C.E]** ^a^ What are the biggest challenges of your job? What are the most rewarding and exciting aspects of your job? What is the reaction of your friends and family to your job?
**Shelter goals and practices**
What are your shelter’s main goals? Does your shelter conduct low cost spay-neuter for street dogs that come into the shelter? **[C.E]** What are current challenges with the work your organization does? In your opinion, how different are the goals of Western animal NGOs from Indian animal NGOs? What changes would you like to see in your organization or Indian animal NGOs as a whole?
**Perceptions of animal welfare**
Do you feed community dogs/free-ranging dogs in your neighborhood? **[C.E]** In a ‘perfect world’, what would the lives of these dogs be like? Do you think we should attempt to get all dogs off the streets into homes or can street dogs have a good quality of life if numbers are controlled? **[C.E]**

^a^**[C.E]**: close ended question.

**Table 4:** Shelter challenges and resiliency factors.

Section 3.1	Challenges	
	Themes	Sub-Themes
Section 3.1.1	High intake	A. Pet abandonment B. Animal overpopulation C. Seasonal fluctuations D. Animal death
Section 3.1.2	Inadequate funding	A. Lack of government support B. Government policy C. Cultural and religious beliefs
Section 3.1.3	Community conflict	A. Rescuer pressure B. Resident pushback C. Incorrect community care
**Section 3.2**	**Resiliency Factors**	
	**Themes**	**Sub-Themes**
Section 3.2.1	Flexibility and prioritization	
Section 3.2.2	Co-worker support	A. Collaboration B. Equity and safe space
Section 3.2.3	Duty of care	
Section 3.2.4	Understanding animal needs	A. Unrestricted movement B. Autonomy C. Community care

## Data Availability

The data presented in this study are available on request from the corresponding author. The data are not publicly available due to the need to protect anonymity of the participants.
